# Occupational Lead Exposure and Associations with Selected Cancers: The Shanghai Men’s and Women’s Health Study Cohorts

**DOI:** 10.1289/ehp.1408171

**Published:** 2015-06-19

**Authors:** Linda M. Liao, Melissa C. Friesen, Yong-Bing Xiang, Hui Cai, Dong-Hee Koh, Bu-Tian Ji, Gong Yang, Hong-Lan Li, Sarah J. Locke, Nathaniel Rothman, Wei Zheng, Yu-Tang Gao, Xiao-Ou Shu, Mark P. Purdue

**Affiliations:** 1 Division of Cancer Epidemiology and Genetics, National Cancer Institute, National Institutes of Health, Department of Health and Human Services, Bethesda, Maryland, USA; 2 Department of Epidemiology, Shanghai Cancer Institute, Shanghai, China; 3 Division of Epidemiology, Department of Medicine, School of Medicine, Vanderbilt University, Nashville, Tennessee, USA; 4 National Cancer Control Institute, National Cancer Center, Goyang, Korea

## Abstract

**Background:**

Epidemiologic studies of occupational lead exposure have suggested increased risks of cancers of the stomach, lung, kidney, brain, and meninges; however, the totality of the evidence is inconsistent.

**Objective:**

We investigated the relationship between occupational lead exposure and cancer incidence at the five abovementioned sites in two prospective cohorts in Shanghai, China.

**Methods:**

Annual job/industry-specific estimates of lead fume and lead dust exposure, derived from a statistical model combining expert lead intensity ratings with inspection measurements, were applied to the lifetime work histories of participants from the Shanghai Women’s Health Study (SWHS; *n* = 73,363) and the Shanghai Men’s Health Study (SMHS; *n* = 61,379) to estimate cumulative exposure to lead fume and lead dust. These metrics were then combined into an overall occupational lead exposure variable. Cohort-specific relative hazard rate ratios (RRs) and 95% confidence intervals (CIs) comparing exposed and unexposed participants were estimated using Cox proportional hazards regression and combined by meta-analysis.

**Results:**

The proportions of SWHS and SMHS participants with estimated occupational lead exposure were 8.9% and 6.9%, respectively. Lead exposure was positively associated with meningioma risk in women only (*n* = 38 unexposed and 9 exposed cases; RR = 2.4; 95% CI: 1.1, 5.0), particularly with above-median cumulative exposure (RR = 3.1; 95% CI: 1.3, 7.4). However, all 12 meningioma cases among men were classified as unexposed to lead. We also observed non-significant associations with lead exposure for cancers of the kidney (*n* = 157 unexposed and 17 ever exposed cases; RR = 1.4; 95% CI: 0.9, 2.3) and brain (*n* = 67 unexposed and 10 ever exposed cases; RR = 1.8; 95% CI: 0.7, 4.8) overall.

**Conclusions:**

Our findings, though limited by small numbers of cases, suggest that lead is associated with the risk of several cancers in women and men.

**Citation:**

Liao LM, Friesen MC, Xiang YB, Cai H, Koh DH, Ji BT, Yang G, Li HL, Locke SJ, Rothman N, Zheng W, Gao YT, Shu XO, Purdue MP. 2016. Occupational lead exposure and associations with selected cancers: the Shanghai Men’s and Women’s Health Study cohorts. Environ Health Perspect 124:97–103; http://dx.doi.org/10.1289/ehp.1408171

## Introduction

Lead is a metal that is commonly used in many industrial settings worldwide, and it is an important environmental pollutant. The occurrence of lead in the environment has decreased greatly in recent decades because of the elimination of most leaded gasoline; however, occupational exposures continue primarily via lead in the storage battery industry and lead pigments in paints [International Agency for Research on Cancer (IARC) 2006]. Occupations that have had frequent high exposures include battery-production workers, battery-recycling workers, foundry workers, lead chemical workers, lead smelter and refinery workers, leaded-glass workers, pigment workers, construction workers, and radiator-repair workers. In most developed countries, strict controls have reduced environmental and occupational exposures to lead; however, lead exposure continues to be an issue in developing countries with rapid industrialization, such as China (Gottesfeld and Pokhrel 2011; IARC 2006). Lead as a gasoline additive is a large contributor to environmental lead exposure, and developing countries, such as China, have generally lagged behind developed countries in banning lead in gasoline. Leaded gasoline was eventually banned in 1999 in China and was gradually phased out over the 2000s; however, other environmental sources continue to contribute to lead exposure in China. The occupational exposure limit (OEL) for lead and inorganic compounds of lead was set in China in 1979 and was based on maximum allowable concentrations of 0.05 mg/m^3^ for lead dust and 0.03 mg/m^3^ for lead fume (Liang et al. 1995). The OELs remained at the same levels but were based on time-weighted averages from 2002 onward, similar to the exposure standards for lead in the United States.

High lead exposure is known to be harmful, particularly for children; established health effects include damage to the brain and nervous system, gastrointestinal problems, anemia, liver and kidney damage, fertility problems, and developmental delays (Abadin et al. 2007). Lead is also a suspected carcinogen, with inorganic lead compounds currently designated by the IARC as probably carcinogenic (Group 2A) based on limited evidence in humans and sufficient evidence in animals (IARC 2006). Organic lead compounds were designated by the IARC as not classifiable with regard to carcinogenicity (Group 3) owing to inadequate evidence. Epidemiologic evidence for carcinogenicity in workers exposed to inorganic lead suggests associations with cancers of the stomach, lung, kidney, brain, and meninges, although the totality of the evidence is inconsistent (IARC 2006; Rousseau et al. 2007; Steenland and Boffetta 2000). Very few previous studies have evaluated occupational lead exposure among women, although differences between the sexes have been observed for lead exposure and metabolism (Vahter et al. 2007). There is thus a need for additional well-designed epidemiologic studies including both men and women to resolve the question of whether lead is a carcinogen (Ward et al. 2010). To that end, we investigated the association between occupational lead exposure and risk of cancers of the stomach, lung, kidney, brain, and meninges in two large prospective cohort studies of women and men in Shanghai, China.

## Methods

*Study population.* The Shanghai Women’s Health Study (SWHS) and the Shanghai Men’s Health Study (SMHS) are two population-based prospective cohort studies based in Shanghai, China. The rationale, design, and methods of both studies have been described in detail previously (Shu et al. 2015; Zheng et al. 2005). Briefly, using a roster provided by the community office, 81,170 permanent female residents 40–70 years of age were approached for the SWHS study between 1996 and 2000 (Zheng et al. 2005), and 83,033 permanent male residents 40–74 years of age were approached for the SMHS between 2002 and 2006 (Shu et al. 2015). Of the 81,170 eligible women, 75,221 participated in the SWHS study, for an overall response rate of 92.7%. It was determined afterwards that 279 of these women did not meet the age eligibility requirements and were excluded, resulting in a cohort of 74,941 women. Of the 83,033 eligible men, 61,480 participated in the SMHS study, for an overall response rate of 74.0%. An additional 14 men were lost to follow-up, resulting in a cohort of 61,466 men. An additional exclusion criterion of prevalent cancers at baseline was also applied (*n* = 1,578 women and *n* = 0 men; having a prior history of cancer was among the exclusion criteria for participation in the SMHS), leaving 73,363 women and 61,466 men in the present analysis. In-person interviews were administered at baseline to obtain information on demographics, lifestyle and dietary habits, medical history, and other characteristics, including lifetime occupational history. All study participants provided written informed consent before being interviewed, and the study protocols were approved by the institutional review boards of all participating institutions (National Cancer Institute, Vanderbilt University and the Shanghai Cancer Institute).

Cohort members are followed for cancer occurrence through in-person follow-up surveys administered every 2–3 years and annual record linkage with the Shanghai Cancer Registry and Vital Statistics Unit. For the SWHS, the response rates for follow-up (i.e., the number of responders/number of surviving cohort members) for the first (2000–2002), second (2002–2004), third (2004–2007), and fourth (2008–2011) in-person follow-up surveys were 99.8%, 98.7%, 96.7%, and 92.0%, respectively. For the SMHS, the response rates for the first (2004–2008) and second (2008–2011) follow-up surveys were 97.6% and 93.6%, respectively. Cohort members known to have permanently moved out of Shanghai or who cannot be contacted in three consecutive follow-ups are considered lost to follow-up. All cancer diagnoses are verified through home visits and medical chart review to ensure pathological confirmation. The cancer sites of interest for this project were stomach cancer [*International Classification of Diseases, 9th Revision* (ICD-9) codes 151.0–151.9], lung cancer (ICD-9 codes 162.0–162.9), kidney cancer (ICD-9 code 189.0), brain cancer (ICD-9 code 191), and meningioma [ICD-9 codes 192.1, 192.3, and 225.2; *International Classification of Diseases for Oncology, 3rd Edition* (ICDO-3) codes 9530–9539]. First-incident cancers of each site of interest were identified through 31 December 2009 in SWHS and 31 December 2010 in SMHS.

*Lead exposure assessment.* Study participants provided a lifetime occupational history, which included all jobs held for at least 1 year, with specific details on job title, type of business, factory name, description of work tasks, and employment dates. Occupational history records were then assigned job and industry codes based on the Standard Chinese Classification of Industries and Occupations for the Third National Population Census of 1982 [China Statistics Archives (CSA) at the University of Illinois at Chicago and China Statistical Information and Consultancy Service Center (CSICSC) 1989].

Lead fume and lead dust were estimated and evaluated separately because they can vary in their particle size and composition and thus may have differing bioavailability and health effects. Lead fume is created by high-temperature processes that form fine particulate through condensation of airborne lead vapor, whereas lead dust is formed from mechanical processes that develop both fine and large particles (Needleman 1992). Because both are measured using the same sampling and analytical processes, they were distinguished here by using industrial hygienists’ judgment of the expected form of lead exposure based on the work activities and lead source. The lead fume and lead dust measures described below do not differentiate lead exposures based on the chemical form (metallic, inorganic, organic) or solubility, which will vary by lead source.

Details on the development of job/industry-specific estimates of exposure to lead fume and lead dust have been previously reported for SWHS and were used for both cohorts in this analysis (Koh et al. 2014). In brief, population-based job exposure matrices (JEMs) were developed to provide expert-based estimates of the probability and intensity of exposure to lead fume and lead dust for the job and industry codes reported in the study participants’ occupational histories. Separate mixed-effects models were developed for lead fume and lead dust to combine the expert ratings of the respective lead intensity metric with its associated inspection measurements (20,084 lead fume measurements; 5,383 lead dust measurements) collected by the Shanghai Center for Disease Control and Prevention between 1954 and 2000. Annual job/industry-specific estimates of lead fume and lead dust exposure were calculated from the fixed-effects terms for the JEM intensity ratings and calendar year and from the random-effects terms for job and industry from their respective mixed models. Job/industry-specific estimates were calculated only for job/industry combinations that met a strict exposure definition based on the JEM probability ratings (job probability = high or industry probability = high and job probability = low, medium, or high); all other job/industry combinations were assigned 0 exposure. We chose this exposure definition to emphasize specificity over sensitivity, as recommended by Kromhout and Vermeulen (2001) for rare exposures in order to minimize bias from exposure misclassification. The lead fume and lead dust models were applied to both cohorts to estimate annual occupational lead fume and lead dust exposure for each study participant; the annual estimates were then summed over each participant’s working life to obtain separate cumulative exposure estimates for lead fume and for lead dust. Median values for cumulative lead fume (0.33 mg/m^3^-years) and lead dust (1.32 mg/m^3^-years) were determined by the combined distribution of the exposed participants across both cohorts. Because of the small numbers, the cumulative estimates of occupational lead fume and lead dust exposure were also combined into an overall lead exposure variable. Subjects’ exposures were further categorized as “never” when no cumulative lead dust or lead fume exposure was assigned, “low” when cumulative exposures for either lead dust and/or lead fume ≤ median and neither lead dust and/or lead fume was > median, and “high” when cumulative exposure > median for either lead dust or lead fume (Table 1). Cumulative lead estimates incorporating 10- and 20-year lags were also constructed. Using the same mixed-effects models, calibrated JEM estimates that used the fixed-effects terms but not the random-effects terms were also calculated and applied to both cohorts; however, we determined that this alternate estimate of cumulative exposure was essentially collinear with the job/industry-specific estimates (Pearson correlation = 0.94 for lead fume and Pearson correlation = 0.99 for lead dust). Thus, we chose to present the more refined job/industry-specific estimates of lead exposure (Koh et al. 2014). We refer the reader to the paper by Koh et al. (2014) for a more detailed discussion of the models and a review of the sensitivity analyses conducted.

**Table 1:** Method for assigning categories of total lead exposure (never, low, high) from estimates of lead dust and lead fume exposure.

Combined lead metric		Lead fume		
		0	≤ Median^*a*^	> Median^*a*^
Lead dust	0	Never	Low	High
	≤ Median^*a*^	Low	Low	High
	> Median^*a*^	High	High	High
^***a***^Median values for cumulative lead fume (0.33 mg/m^3^-years) and lead dust (1.32 mg/m^3^-years) were determined by the combined distribution of the exposed participants across both cohorts.				

*Statistical analyses.* Cox proportional hazards regression, with age as the time scale, was used to estimate cohort-specific hazard rate ratios (RRs) and 95% confidence intervals (CIs) for the association between lead exposure and risk of cancer at each site with adjustment for potential confounders: education level (elementary school or less, middle school, high school, and professional education/college or higher), family income level (study-specific, see Table 2 for definition), lifetime pack-years of cigarette use (study-specific—SWHS: never smoker, former smoker ≤ 7.4 pack-years, former smoker > 7.4 pack-years, current smoker ≤ 7.4 pack-years, current smoker > 7.4 pack-years; SMHS: never smoker, former smoker ≤ 22.2 pack-years, former smoker > 22.2 pack-years, current smoker ≤ 22.5 pack-years, current smoker > 22.5 pack-years), and menopause status (defined as absence of menstruation for ≥ 12 months; SWHS only). All confounders were baseline characteristics. Models additionally adjusted for body mass index (continuous) and alcohol consumption (continuous) yielded virtually identical results and are not presented here. Study participants with missing data (no occupational history provided) were treated as a separate category in the analysis. We then calculated summary RR estimates from cohort-specific results through meta-analysis using a random-effects model. We tested for potential RR heterogeneity between cohorts using Cochran’s *Q* statistic. Cohort-specific analyses were conducted using SAS, version 9.3 (SAS Institute Inc., Cary, NC), and the meta-analysis was conducted using STATA, version 13.0 (StataCorp LP, College Station, TX).

**Table 2:** Selected characteristics of the Shanghai Women’s Health Study (SWHS) and the Shanghai Men’s Health Study (SMHS) cohorts.

Characteristics^*a*^	SWHS (*n* = 73,363)	SMHS (*n* = 61,466)
Age at baseline (years); mean (range)	52.0 (40–70)	55.4 (40–75)
Education
Elementary school or less;* n *(%)	15,687 (21.4)	4,083 (6.7)
Middle school;* n *(percent)	27,270 (37.2)	20,330 (33.5)
High school;* n *(percent)	20,490 (27.9)	21,856 (36.1)
Professional education/college or higher;* n *(%)	9,903 (13.5)	14,334 (23.7)
Income^*b*^
Low;* n *(%)	11,813 (16.1)	33,845 (55.2)
Lower middle;* n*(%)	28,063 (38.3)	21,539 (35.1)
Upper middle;* n *(%)	20,599 (28.1)	4,597 (7.5)
High;* n *(%)	12,872 (17.6)	1,358 (2.2)
Occupation^*c*^
Professional, administrator;* n *(%)	21,026 (28.8)	16,308 (26.6)
Clerical worker;* n *(%)	15,198 (20.8)	13,469 (21.9)
Manual laborer;* n *(%)	36,862 (50.4)	31,619 (51.5)
Lifetime pack-years of cigarette use^*d*^
Never;* n *(%)	71,320 (97.2)	18,669 (30.4)
Former–low;* n *(%)	158 (0.2)	3,689 (6.0)
Former–high;* n *(%)	141 (0.2)	3,065 (5.0)
Current–low;* n *(%)	875 (1.2)	18,012 (29.3)
Current–high;* n *(%)	868 (1.2)	18,024 (29.3)
Alcohol consumption
Ever;* n *(%)	1,654 (2.3)	20,728 (33.7)
Grams per day; mean (range)	9.1 (0–150)	11.8 (0–608)
Body mass index (kg/m^2^); mean (range)	24.0 (13–49)	23.7 (12–40)
Menopausal status
Premenopausal;* n *(%)	37,457 (51.1)	NA
Postmenopausal;* n *(%)	35,891 (48.9)	NA
Lead dust
Never;* n *(%)	70,378 (95.9)	57,241 (93.2)
Ever;* n *(%)	2,709 (3.7)	4,138 (6.7)
Year first exposed among exposed participants; median (range)^*e*^	1972 (1941–1999)	1976 (1943–2005)
Cumulative exposure (mg/m^3^-year) among exposed participants; median (range)^*e*^	1.56 (0.01–11.2)	1.03 (0.006–7.8)
Annual exposure of exposed participants by time period (mg/m^3^); median (range)^*e*^
1930–1959	0.15 (0.04–0.52)	0.11 (0.02–0.30)
1960s	0.14 (0.03–0.52)	0.10 (0.02–0.30)
1970s	0.10 (0.03–0.37)	0.07 (0.01–0.21)
1980s	0.11 (0.03–0.42)	0.08 (0.01–0.24)
1990s	0.056 (0.010–0.32)	0.04 (0.003–0.18)
2000s	0.012 (0.008–0.030)	0.009 (0.002–0.025)
Lead fume
Never;* n *(%)	67,280 (91.7)	59,962 (97.6)
Ever;* n *(%)	5,807 (7.9)	1,417 (2.3)
Year first exposed among exposed participants; median (range)^*e*^	1972 (1945–1999)	1975 (1941–2005)
Cumulative exposure (mg/m^3^-year) among exposed participants; median (range)^*e*^	0.29 (0.003–6.6)	0.46 (0.001–11.0)
Annual exposure of exposed participants by time period (mg/m^3^); median (range)^*e*^
1930–1959	0.028 (0.015–0.24)	0.063 (0.009–0.33)
1960s	0.025 (0.014–0.24)	0.049 (0.008–0.33)
1970s	0.020 (0.013–0.22)	0.038 (0.005–0.28)
1980s	0.020 (0.005–0.24)	0.30 (0.003–0.30)
1990s	0.005 (0.002–0.080)	0.008 (0.001–0.038)
2000s	0.003 (0.001–0.019)	0.004 (0.001–0.038)
Lead dust and fume
Never;* n *(%)	66,813 (91.1)	57,123 (93.0)
Ever;* n *(%)	6,274 (8.9)	4,256 (6.9)
Year first exposed; median (range)^*e*^	1972 (1941–1999)	1975 (1941–2005)
Years of follow-up; mean (range)	10.8 (0.1–13)	6.4 (0.1–9)
NA, not applicable. ^***a***^Number of subjects with missing data noted for education (*n *= 13 women, 863 men); income (*n *= 16 women, 127 men); occupation (*n *= 277 women, 70 men); smoking (*n *= 1 woman, 7 men); alcohol consumption (*n *= 0 women, 1 man); menopausal status (*n *= 15 women); lead dust and/or lead fume exposure data (*n *= 276 women, 87 men). ^***b***^Income cutpoints were as follows: SWHS: < 10,000 (low), 10,000 to < 20,000 (lower middle), 20,000 to < 30,000 (upper middle), and ≥ 30,000 (high) yuan/year per household. SMHS: < 1,000 (low), 1,000 to < 2,000 (lower middle), 2,000 to < 3,000 (upper middle), and ≥ 3,000 (high) yuan/month. ^***c***^Longest occupation reported during occupational history. ^***d***^Lifetime pack-years of cigarette use cutpoints were as follows: SWHS: never smoker, former–low smoker ≤ 7.4 pack-years, former–high smoker > 7.4 pack-years, current–low smoker ≤ 7.4 pack-years, current–high smoker > 7.4 pack-years; SMHS: never smoker, former–low smoker ≤ 22.2 pack-years, former–high smoker > 22.2 pack-years, current–low smoker ≤ 22.5 pack-years, current–high smoker > 22.5 pack-years. ^***e***^Results presented are for exposed subjects only.		

## Results

The SWHS and SMHS analytic cohorts included 73,363 women (mean follow-up, 10.8 years) and 61,466 men (mean follow-up, 6.4 years), respectively. SMHS participants tended to report a higher level of education than SWHS participants but reported lower household income (Table 2). Smoking and alcohol consumption were much more common in the male cohort than in the female cohort. In both cohorts, approximately half of women (50.4%) and men (51.5%) reported working as manual laborers as their longest occupation during their occupational history at baseline. Overall, the proportion of study participants (women and men combined) identified as ever exposed to lead fume was 7.4%, and the proportion of those identified as ever exposed to lead dust was 3.1%. Lead exposure concentrations in these cohorts decreased considerably from 1965 to 2000 (Table 2) (Koh et al. 2014). The exposure prevalence was slightly higher in the female cohort (lead fume, 7.9%; lead dust, 3.7%) than in the male cohort (2.3%; 6.7%). The median first year of lead fume or lead dust exposure in the female cohort was slightly earlier than that in the male cohort (1972 vs. 1975).

The three most commonly reported jobs (based on number of exposed person-years) that were exposed to lead fume or lead dust in the female cohort (“Install/assemble electric/electronic equipment,” 42.1% of exposed person-years; “Welders,” 11.4%; “Other electric/electronic equipment install/maintenance,” 7.9%) showed a different pattern than those reported in the SMHS cohort (“Install/assemble electric/electronic equipment,” 14.2%; “Rolling mill & machinery operators,” 12.0%; “Smelters,” 8.4%) (see Supplemental Material, Table S1). Five of the top 10 jobs exposed to lead fume or lead dust were the same in both cohorts. Males, however, appeared to be exposed to lead through a wider range of occupations than females; the 10 most commonly exposed occupations reported by males accounted for only 68% of exposed person-years versus 83% among females.

A total of 1,918 incident first-primary cancers of interest were identified during follow-up in the two cohorts, including 634 stomach cancers, 974 lung cancers, 174 kidney cancers, 77 brain cancers, and 59 meningiomas (47 females and 12 males). We observed non-significant associations with ever exposure to lead dust or lead fume for cancers of the kidney (*n* = 157 unexposed and 17 ever exposed cases; RR = 1.4; 95% CI: 0.9, 2.3) and brain (*n* = 67 unexposed and 10 ever exposed cases; RR = 1.8; 95% CI: 0.7, 4.8) (Table 3). A further elevated risk of kidney cancer was observed among those with high cumulative exposure to lead based on the combined lead metric (*n* = 12 exposed cases; RR = 1.8; 95% CI: 1.3, 7.4). There was no clear dose–response association with brain cancer because the association was null among those with high exposure. Similar results for kidney and brain cancers were observed for separate analyses of lead dust and lead fume. In our cohort of females, an elevated risk with ever exposure to lead was observed for meningioma (*n* = 38 unexposed cases and 9 ever exposed cases; RR = 2.4; 95% CI: 1.1, 5.0; Table 3). This association was particularly strong for high cumulative lead exposure based on the combined lead metric (*n* = 38 unexposed cases and 6 exposed cases; RR = 3.1; 95% CI: 1.3, 7.4) and was also present in separate analyses of both lead dust and lead fume. We could not compute an association with meningioma in the male cohort because none of the 12 cases was assessed as having lead exposure. Although a hazard ratio for meningioma was not estimable for the male cohort, given the lack of exposed male cases, it is unlikely that lead exposure was positively associated with risk in this study population (see Supplemental Material, Tables S2 and S3).

**Table 3:** Meta-analysis summary estimates for associations between lead and cancer.

Lead exposure	Kidney		Lung		Stomach		Brain		Meningioma (SWHS only)^*a*^	
	Cases (*n*)	RR (95% CI)^*b*^	Cases (*n*)	RR (95% CI)	Cases (*n*)	RR (95% CI)	Cases (*n*)	RR (95% CI)	Cases (*n*)	RR (95% CI)
Lead dust^*c*^
Never	168	1.0	948	1.0	619	1.0	72	1.0	42	1.0
Ever	6	1.3 (0.6, 2.8)	26	0.9 (0.6, 1.4)	15	0.8 (0.4, 1.4)	5	2.3 (0.9, 5.8)	5	2.9 (1.1, 7.3)
Low	1	0.8 (0.1, 6.0)	13	0.9 (0.3, 3.1)	7	0.8 (0.4, 1.7)	2	2.0 (0.5, 8.3)	1	1.5 (0.2, 10.6)
High	5	2.3 (0.8, 6.7)	13	0.8 (0.5, 1.3)	8	0.8 (0.4, 1.7)	3	2.6 (0.8, 8.2)	4	3.8 (1.4, 10.7)
Lead fume^*c*^
Never	157	1.0	902	1.0	587	1.0	68	1.0	38	1.0
Ever	17	1.5 (0.9, 2.5)	72	0.9 (0.5, 1.8)^*d*^	47	1.0 (0.5, 2.0)^*d*^	9	1.8 (0.8, 4.1)	9	2.6 (1.2, 5.4)
Low	6	1.2 (0.4, 3.4)	22	0.8 (0.4, 1.3)	14	0.7 (0.4, 1.2)	6	2.9 (1.2, 6.7)	4	2.2 (0.8, 6.3)
High	11	1.8 (0.9, 3.7)	50	1.1 (0.6, 1.9)	33	1.2 (0.6, 2.4)	3	1.1 (0.3, 3.5)	5	3.0 (1.2, 7.6)
Combined lead dust and fume^*e*^
Never	157	1.0	900	1.0	585	1.0	67	1.0	38	1.0
Ever	17	1.4 (0.9, 2.3)	74	0.9 (0.5, 1.7)^*d*^	49	1.0 (0.5, 1.9)^*d*^	10	1.8 (0.7, 4.8)	9	2.4 (1.1, 5.0)
Low	5	1.0 (0.4, 2.5)	22	0.7 (0.4, 1.2)	14	0.7 (0.4, 1.2)	7	3.1 (1.0, 9.1)	3	1.7 (0.5, 5.4)
High	12	1.8 (1.0, 3.3)	52	1.1 (0.6, 1.9)	35	1.2 (0.6, 2.2)	3	1.0 (0.3, 3.2)	6	3.1 (1.3, 7.4)
Abbreviations: RR, relative hazard rate ratio; CI, confidence interval. ^***a***^Meningioma results are only from the SWHS cohort because there were no exposed meningioma cases (*n *= 12) in the SMHS cohort. ^***b***^Adjusted for education, income level, lifetime pack-years of cigarette use, and menopause status (women only). ^***c***^Levels of lead exposure: low: ≤ median; high: > median. ^***d***^Test for heterogeneity (Cochran’s Q statistic) indicated heterogeneity at *p* < 0.05. ^***e***^Levels of combined lead exposure: low: exposure to lead dust or lead fume, but not high exposure (≤ median) to either; high: at least one high exposure (> median) to lead dust or lead fume.										

We observed null findings for cancers of the lung and stomach (Table 3); however, there was evidence of heterogeneity between cohorts that could be partly due to the lack of an association in the female cohort. When the cohorts were analyzed separately (Table 4), high cumulative lead exposure based on the combined lead metric was non-significantly associated with the risk of lung cancer in the male cohort (*n* = 460 unexposed cases and 35 exposed cases; RR = 1.4; 95% CI: 0.98, 2.0) but not the female cohort (*n* = 440 unexposed cases and 17 exposed cases; RR = 0.8; 95% CI: 0.5, 1.3; high exposure heterogeneity *p*-value = 0.06). Similarly, a suggestive elevated risk of stomach cancer was associated with high lead exposure based on the combined lead metric (*n* = 293 unexposed cases and 23 exposed cases; RR = 1.6; 95% CI: 1.0, 2.4) in the male cohort but was not observed in the female cohort (*n* = 292 unexposed cases and 12 exposed cases; RR = 0.8; 95% CI: 0.5, 1.5; high exposure heterogeneity *p*-value = 0.07). Associations of lung and stomach cancer with high (> median) exposure in the male cohort were stronger for lead fume than for lead dust (see Supplemental Material, Table S3). It should also be noted that males were more likely to be exposed to lead fume than to lead dust based upon the number of exposed cases.

**Table 4:** Cohort-specific associations between lead exposure and cancer risk.

Combined lead dust and lead fume^*b*^	Kidney				Lung				Stomach				Brain			
	SWHS		SMHS		SWHS		SMHS		SWHS		SMHS		SWHS		SMHS	
	Cases (*n*)	RR (95% CI)^*a*^	Cases (*n*)	RR (95% CI)	Cases (*n*)	RR (95% CI)	Cases (*n*)	RR (95% CI)	Cases (*n*)	RR (95% CI)	Cases (*n*)	RR (95% CI)	Cases (*n*)	RR (95% CI)	Cases (*n*)	RR (95% CI)
Never	76	1.0	81	1.0	440	1.0	460	1.0	292	1.0	293	1.0	34	1.0	33	1.0
Ever	8	1.3 (0.6, 2.6)	9	1.6 (0.8, 3.1)	27	0.7 (0.5, 1.0)	47	1.2 (0.9, 1.7)	19	0.7 (0.4, 1.1)	30	1.4 (0.9, 2.0)	8	2.6 (1.2, 5.6)	2	0.9 (0.2, 3.8)
Low	4	1.3 (0.5, 3.5)	1	0.5 (0.1, 3.2)	10	0.6 (0.3, 1.1)	12	1.0 (0.5, 1.7)	7	0.6 (0.3, 1.2)	7	0.9 (0.4, 1.9)	6	4.2 (1.8, 10.1)	1	1.2 (0.2, 8.5)
High	4	1.2 (0.5, 3.4)	8	2.3 (1.1, 4.7)	17	0.8 (0.5, 1.3)	35	1.4 (0.98, 2.0)	12	0.8 (0.5, 1.5)	23	1.6 (1.03, 2.4)	2	1.2 (0.3, 5.0)	1	0.7 (0.1, 5.4)
Abbreviations: RR, relative hazard rate ratio; CI, confidence interval. ^***a***^Adjusted for education, income level, lifetime pack-years of cigarette use, and menopause status (women only). ^***b***^Levels of lead exposure: low: exposed to lead dust or lead fume, but not high exposure (≤ median) to either; high: at least one high exposure (> median) to lead dust or lead fume.																

Evaluations of lead exposure with 10- and 20-year time lags were generally consistent with our risk estimates and did not change our findings (see Supplemental Material, Tables S2 and S3). The lack of change in our risk estimates is consistent with the trend described by Koh et al. (2014) of lead fume and lead dust concentrations declining over time in this population. Thus, excluding recent lead exposure had little impact on risk estimates and further demonstrates that most lead exposure occurred in the distant past in these cohorts.

## Discussion

We found evidence of an association between exposure to lead dust or lead fume and an increased subsequent risk of meningioma in the female cohort, with higher cumulative exposure associated with higher risk. We were unable to evaluate this association in the male cohort owing to a lack of exposed male cases, but it is unlikely that lead exposure was positively associated with meningioma risk in the male cohort. Nonsignificant associations with lead exposure were observed overall for cancers of the kidney and brain. The association with brain cancer appeared to be limited to the female cohort. In addition, elevated risks of lung and stomach cancer were observed with high lead exposure in the male cohort, but no such associations were observed in the female cohort. Our findings suggest that lead is associated with the risk of several cancers, but these findings are limited by small numbers of cases, particularly for kidney and brain cancer.

In 2006, when the IARC classified lead as a probable carcinogen, the epidemiological evidence was the most consistent for stomach cancer, with elevated kidney and lung cancer risks observed in some but not all studies (IARC 2006). Evidence from human studies was considered limited; thus, several studies conducted since the publication of the IARC monograph have attempted to further evaluate the risks for these cancers. Some of these investigations have reported associations with meningioma and brain cancer that are consistent with our findings. A case–control study of death certificate data among U.S. women observed an association between jobs involving occupational lead exposure and risk of meningioma (*n* = 161 cases) (Cocco et al. 1999). A more recent U.S. case–control study (*n* = 197 meningiomas) found evidence of an association between cumulative lead exposure and meningioma risk among males but not among females; however, the authors observed a consistently increased risk of meningioma among a subset of lead-exposed individuals with susceptible genotypes of *ALAD2*, a gene influencing lead bioavailability (Bhatti et al. 2009; Rajaraman et al. 2006). Navas-Acién et al. (2002) reported an increased relative risk of meningioma with occupational lead exposure among Swedish males (*n* = 848 meningiomas), but there was an insufficient number of exposed females in their cohort study to allow a risk estimate to be computed. An increased relative risk of brain and central nervous system cancers (including 298 meningiomas) associated with high levels of occupational lead exposure was reported in an occupational cohort of Finnish women (Wesseling et al. 2002). Findings from several case–control (Cocco et al. 1998) and cohort (Anttila et al. 1996; Ilychova and Zaridze 2012; van Wijngaarden and Dosemeci 2006; Wesseling et al. 2002) studies suggest an association between lead and brain cancer.

We also observed associations between lead exposure and an increased risk of kidney cancer, primarily in the male cohort, and increased risks of lung and stomach cancer in the male cohort only. Findings from other recent epidemiological studies, few of which included women, have been inconsistent for these cancers. A multicenter case–control study of both men and women conducted in central and eastern Europe observed an elevated risk of renal cell carcinoma (RCC) with high occupational lead exposure (Boffetta et al. 2011). A subsequent analysis from this study further reported that genetic variation in *ALAD* may modify the lead–RCC association (van Bemmel et al. 2011). Southard et al. conducted a nested case–control study of Finnish male smokers and observed an increased relative risk of RCC (odds ratio = 2.0; 95% CI: 1.0, 3.9) with higher blood lead concentrations (Southard et al. 2012). Elevated blood lead concentration was also associated with increased lung cancer risk in a separate Finnish cohort (Anttila et al. 1995). In contrast, a retrospective cohort study of male workers in Australia who were exposed to lead did not find an increased risk of any cancers previously linked to lead exposure (Gwini et al. 2012). Rousseau et al. (2007) evaluated associations between the risk of 11 types of cancer and lead exposure in a case–control study of men in Canada and only found an association between organic lead exposure and stomach cancer. In a subsequent study, Wynant et al. (2013) did not find an association between lead exposure and lung cancer in a pooled analysis that included the previous study. Among males, we observed stronger associations with lung and stomach cancer for the lead fume metrics than for the lead dust metrics. Exposure to lead fume was more common than exposure to lead dust among study participants overall. Previous studies did not discern between the two lead measures; however, it is possible that the finer particulate matter produced by lead fume is more readily absorbed via inhalation or ingestion than lead dust (National Toxicology Program 2011).

The mechanisms by which lead may increase cancer risk remain unclear. Inhalation and oral ingestion are the two primary routes through which lead enters the body. Thus, both the lungs and stomach are organs that come into initial contact with lead when exposure occurs, but lead can enter the bloodstream and affect other organs. The brain and nervous system are especially sensitive to the potential toxic effects of lead owing to its ability to pass through the blood–brain barrier (Inskip et al. 1995). The high reabsorptive activity of the renal proximal tubules also lends itself to the accumulation and uptake of lead in the kidney (IARC 2006). It has been suggested that lead may act through indirect mechanisms to facilitate the carcinogenic effects of other DNA-damaging agents because experimental studies have shown very little or no mutagenicity for the main forms of lead (IARC 2006; Winder and Bonin 1993). Therefore, it has been proposed that lead may play a role in carcinogenesis through mechanisms that involve oxidative damage, induction of apoptosis, altered cell-signaling pathways, inhibition of DNA synthesis and repair of damage, and interaction with DNA-binding proteins (IARC 2006; Restrepo et al. 2000; Silbergeld 2003).

Differential associations by cohort/sex were observed between lead exposure and several cancer sites. There are several potential explanations for the observed differences. Sex-related differences in lead metabolism may have played a role in the differential associations that we observed (Björkman et al. 2000; Vahter et al. 2007). The different patterns of lead-exposed occupations reported for males and females may have led to differences by cohort in workplace lead exposure intensity and duration that were not captured by our exposure assessment. Although the most common occupation reported was the same among males and females, this occupation (installing or assembling electronic equipment) accounted for 42% of the exposure among females and only 14% among males. This finding suggests that males were exposed to a wider range of occupational sources of lead than were females. There is also a large difference in smoking prevalence between the two cohorts, although our findings are adjusted for pack-years smoked. We also note that the follow-up period for the male cohort was shorter than that for the female cohort (Table 2). Lastly, we cannot rule out the possibility that our sex-specific findings are due to chance, given that we had small numbers of cases for some cancer sites, or that they are due to residual confounding associated with sex.

Our study has many strengths, including the inclusion of two large population-based prospective cohorts with high response rates at recruitment and follow-up. Both cohorts captured extensive information on potential confounders, allowing us to adjust for potential confounding risk factors, such as smoking, for which industry-based cohorts are rarely able to account. Detailed lifetime occupational histories were collected before cancer development, eliminating the potential for recall bias. Although we combined results from two large cohort studies, our findings were limited by small numbers of exposed cases for some cancer sites, most notably brain cancer and meningioma, and by a relatively short follow-up period for the male cohort, thus limiting our statistical power to detect overall and sex-specific associations and possibly resulting in unstable risk estimates that should be interpreted with caution.

Another strength of our study was its ability to capture important time trends in lead exposure by using estimates based on a framework that calibrated an expert-based JEM using inspection measurements collected from Shanghai-area worksites. As is the case with all JEMs, the exposure assessment approach was unable to account for variations in exposure among workers who worked within a particular occupational-industrial grouping and time period (Koh et al. 2014). Another strength of the exposure assessment was that it distinguished between lead fume and lead dust, which partly captured differences in lead composition and bioavailability. This differentiation can introduce some exposure misclassification because it is based on expert opinion to characterize the lead source from observations of work activities while exposure measurements are collected, and for the JEM, it is based on the expected source and work activities for a given occupation or industry. Further differentiation of lead exposure according to solubility or composition was not possible in this study given the limited occupational information and the wide variety but low prevalence of the lead-exposed occupations and industries represented in these cohorts. In addition, exposure estimates for other metals that may be correlated with occupational lead exposure were not available; as a consequence, we are unable to rule out potential confounding by other metals. With regard to meningioma, there are currently no established associations between other metals and meningioma that would be expected to confound our observed association.

## Conclusion

In conclusion, our findings, though limited by small numbers of cases, suggest that lead exposure is associated with an increased risk of several cancers, in particular, meningioma, brain cancer, and kidney cancer. The associations between lead and meningioma and between lead and brain cancer that we observed among women in our study underscore the importance of including women in future studies evaluating the carcinogenicity of lead.

## Supplemental Material

(336 KB) PDF
