# Supplementary material for: Occupational Lead Exposure and Associations with Selected Cancers: The Shanghai Men’s and Women’s Health Study Cohorts
Source: Environ Health Perspect. 2015 Jun 19;124(1):97–103. doi: 10.1289/ehp.1408171 (PMC4710592; doi:10.1289/ehp.1408171)
Supplement: (336 KB) PDF [file ehp.1408171.s001.acco.pdf]

Environ Health Perspect
DOI: 10.1289/ehp.1408171

Note to Readers: *EHP* strives to ensure that all journal content is accessible to all readers. However, some figures and Supplemental Material published in *EHP* articles may not conform to 508 standards due to the complexity of the information being presented. If you need assistance accessing journal content, please contact ehp508@niehs.nih.gov. Our staff will work with you to assess and meet your accessibility needs within 3 working days.

Supplemental Material

Occupational Lead Exposure and Associations with Selected Cancers: The Shanghai Men's and Women's Health Study Cohorts

Linda M. Liao, Melissa C. Friesen, Yong-Bing Xiang, Hui Cai, Dong-Hee Koh, Bu-Tian Ji, Gong Yang, Hong-Lan Li, Sarah J. Locke, Nathaniel Rothman, Wei Zheng, Yu-Tang Gao, Xiao-Ou Shu, and Mark P. Purdue

Table of Contents

Table S1. Top ten jobs exposed to lead fume or dust in the Shanghai Women's Health Study and the Shanghai Men's Health Study.

Table S2. Relationship between lead and cancer risk in the Shanghai Women's Health Study.

Table S3. Relationship between lead and cancer risk in the Shanghai Men's Health Study.

Table S1. Top ten jobs exposed to lead fume or dust in the Shanghai Women's Health Study and the Shanghai Men's Health Study.

Occupation	N exposed person-years	%
SWHS		
863: Install/Assembly Electric/Electron Equip	43589	42.1
882: Welders	11814	11.4
869: Other Electric/Electron Equip Install/Maint	8208	7.9
961: Examiners/Inspectors (Workers)	4170	4.0
823: Printing Workers	4006	3.9
723: Foundry Workers	3685	3.6
844: Metal-cut Machine Tools, Blade Sharpen	3074	3.0
994: Warehouse Workers	2912	2.8
523: Odds-and-Ends Workers	2198	2.1
093: Accounting Personnel[a]	2094	2.0
		82.9
SMHS		
863: Install/Assemble Electric/Electron Equip	10931	14.2
722: Rolling Mill & Machinery Operators	9227	12.0
721: Smelters	6481	8.4
852: Machinery Maintenance Workers (casting, welding, etc.)	6193	8.0
869: Other Electric/Electron Equip Install/Maint (Lead Battery)	4159	5.4
842: Fitters	3577	4.6
864: Electricians	3449	4.5
844: Metal-cut Machine Tools, Blade Sharpen	3140	4.1
961: Examiners/Inspectors	2825	3.7
994: Warehouse Workers (dust in hump field)	2537	3.3
		68.1

[a] Includes only accounting personnel working in industries with a high probability of lead dust exposure (e.g. smelting/metalworking, electronic assembly). Lead levels for these subjects were estimated to be very low (mean lead fume = <0.001 mg/m^3; mean lead dust = 0 mg/m^3).

Table S2. Relationship between lead and cancer risk in the Shanghai Women's Health Study.

Exposure	Kidney cancer cases (N)	RR (95% CI)[a]	Lung cancer cases (N)	RR (95% CI)[a]	Stomach cancer cases (N)	RR (95% CI)[a]	Brain cancer cases (N)	RR (95% CI)[a]	Meningioma cases (N)	RR (95% CI)[a]
**Ever fume/dust**										
Never	76	1.00	440	1.00	292	1.00	34	1.00	38	1.00
Ever	8	1.25 (0.60, 2.61)	27	0.68 (0.46, 1.00)	19	0.69 (0.43, 1.10)	8	2.58 (1.18, 5.63)	9	2.39 (1.14, 4.98)
Low	4	1.27 (0.46, 3.48)	10	0.56 (0.30, 1.05)	7	0.55 (0.26, 1.17)	6	4.21 (1.75, 10.1)	3	1.66 (0.51, 5.40)
High	4	1.24 (0.45, 3.41)	17	0.78 (0.48, 1.27)	12	0.81 (0.46, 1.45)	2	1.19 (0.28, 4.98)	6	3.07 (1.28, 7.35)
**Ever dust**										
No	81	1.00	455	1.00	304	1.00	39	1.00	42	1.00
Yes	3	1.05 (0.33, 3.33)	12	0.70 (0.39, 1.24)	7	0.57 (0.27, 1.22)	3	1.99 (0.61, 6.47)	5	2.88 (1.13, 7.31)
**Lead Dust estimates**										
No	81	1.00	455	1.00	304	1.00	39	1.00	42	1.00
≤median	1	0.84 (0.12, 6.04)	3	0.46 (0.15, 1.44)	3	0.64 (0.20, 1.98)	1	1.73 (0.24, 12.6)	1	1.46 (0.20, 10.62)
>median	2	1.20 (0.29, 4.89)	9	0.84 (0.43, 1.63)	4	0.54 (0.20, 1.44)	2	2.15 (0.51, 8.97)	4	3.81 (1.35, 10.72)
**Ever dust-10 y lag**										
No	81	1.00	455	1.00	304	1.00	39	1.00	42	1.00
Yes	3	1.05 (0.33, 3.33)	12	0.70 (0.39, 1.24)	7	0.57 (0.27, 1.22)	3	1.99 (0.61, 6.48)	5	2.88 (1.13, 7.31)
**Lead Dust estimates - 10 y lag**										
No	81	1.00	455	1.00	304	1.00	39	1.00		1.00
≤median	2	1.68 (0.41, 6.85)	3	0.46 (0.15, 1.44)	3	0.64 (0.20, 1.98)	1	1.73 (0.24, 12.6)	1	1.46 (0.20, 10.62)
>median	1	0.60 (0.08, 4.30)	9	0.84 (0.43, 1.63)	4	0.54 (0.20, 1.44)	2	2.15 (0.51, 8.97)	4	3.81 (1.35, 10.72)
**Ever dust-20 y lag**										
No	82	1.00	455	1.00	304	1.00	39	1.00	42	1.00
Yes	2	0.72 (0.18, 2.94)	12	0.72 (0.40, 1.28)	7	0.60 (0.28, 1.26)	3	2.07 (0.64, 6.75)	5	3.04 (1.19, 7.72)
**Lead Dust estimates - 20 y lag**										
No	82	1.00	455	1.00	304	1.00	39	1.00	42	1.00
≤median	1	0.84 (0.12, 6.06)	4	0.63 (0.23, 1.68)	3	0.65 (0.21, 2.02)	1	1.76 (0.24, 12.8)	1	1.48 (0.20, 10.77)
>median	1	0.63 (0.09, 4.55)	8	0.78 (0.38, 1.56)	4	0.56 (0.21, 1.51)	2	2.27 (0.54, 9.52)	4	4.14 (1.47, 11.67)

Exposure	Kidney cancer cases (N)	RR (95% CI)[a]	Lung cancer cases (N)	RR (95% CI)[a]	Stomach cancer cases (N)	RR (95% CI)[a]	Brain cancer cases (N)	RR (95% CI)[a]	Meningioma cases (N)	RR (95% CI)[a]
**Ever fume**										
No	76	1.00	442	1.00	294	1.00	35	1.00	38	1.00
Yes	8	1.37 (0.66, 2.86)	25	0.67 (0.45, 1.01)	17	0.66 (0.41, 1.09)	7	2.37 (1.04, 5.40)	9	2.57 (1.23, 5.37)
**Lead Fume estimates**										
No	76	1.00	442	1.00	294	1.00	35	1.00	38	1.00
≤median	5	1.63 (0.65, 4.06)	10	0.56 (0.30, 1.06)	7	0.56 (0.26, 1.19)	5	3.47 (1.34, 8.97)	4	2.21 (0.78, 6.26)
>median	3	1.08 (0.34, 3.46)	15	0.78 (0.46, 1.30)	10	0.76 (0.41, 1.44)	2	1.32 (0.31, 5.55)	5	2.96 (1.15, 7.64)
**Ever fume-10 y lag**										
No	76	1.00	442	1.00	294	1.00	35	1.00	38	1.00
Yes	8	1.37 (0.66, 2.86)	25	0.67 (0.45, 1.01)	17	0.66 (0.41, 1.09)	7	2.37 (1.04, 5.40)	9	2.57 (1.23, 5.37)
**Lead Fume estimates-10 y lag**										
No	76	1.00	442	1.00	294	1.00	35	1.00	38	1.00
≤median	5	1.63 (0.65, 4.06)	10	0.56 (0.30, 1.06)	7	0.56 (0.26, 1.19)	5	3.47 (1.34, 8.97)	4	2.21 (0.78, 6.26)
>median	3	1.08 (0.34, 3.46)	15	0.78 (0.46, 1.30)	10	0.76 (0.41, 1.44)	2	1.32 (0.31, 5.55)	5	2.96 (1.15, 7.64)
**Ever fume-20 y lag**										
No	78	1.00	444	1.00	294	1.00	35	1.00	38	1.00
Yes	6	1.03 (0.45, 2.38)	23	0.63 (0.41, 0.96)	17	0.68 (0.42, 1.12)	7	2.44 (1.07, 5.56)	9	2.68 (1.28, 5.60)
**Lead Fume estimates-20 y lag**										
No	78	1.00	444	1.00	294	1.00	35	1.00	38	1.00
≤median	3	0.97 (0.30, 3.08)	10	0.57 (0.30, 1.07)	8	0.65 (0.32, 1.32)	5	3.52 (1.36, 9.08)	5	2.84 (1.11, 7.29)
>median	3	1.10 (0.34, 3.51)	13	0.69 (0.40, 1.20)	9	0.71 (0.37, 1.39)	2	1.38 (0.33, 5.80)	4	2.50 (0.88, 7.11)

[a]Adjusted for education, income level, lifetime pack-years of cigarette use, and menopause status (women only).

Table S3. Relationship between lead and cancer risk in the Shanghai Men's Health Study.

Exposure	Kidney cancer cases (N)	RR (95% CI)[a]	Lung cancer cases (N)	RR (95% CI)[a]	Stomach cancer cases (N)	RR (95% CI)[a]	Brain cancer cases (N)	RR (95% CI)[a]	Meningioma cases (N)	RR (95% CI)[a]
**Ever fume/dust**										
Never	81	1.00	460	1.00	293	1.00	33	1.00	12	1.00
Ever	9	1.56 (0.78, 3.11)	47	1.24 (0.91, 1.67)	30	1.35 (0.92, 1.96)	2	0.90 (0.22, 3.78)	0	-
Low	1	0.45 (0.06, 3.23)	12	0.95 (0.53, 1.68)	7	0.91 (0.43, 1.92)	1	1.16 (0.16, 8.53)	0	-
High	8	2.26 (1.09, 4.70)	35	1.38 (0.98, 1.95)	23	1.58 (1.03, 2.42)	1	0.74 (0.10, 5.42)	0	-
**Ever dust**										
No	87	1.00	493	1.00	315	1.00	33	1.00	12	1.00
Yes	3	1.50 (0.48, 4.76)	14	1.12 (0.66, 1.91)	8	1.06 (0.52, 2.14)	2	2.89 (0.69, 12.1)	0	-
**Lead Dust estimates**										
No	87	1.00	493	1.00	315	1.00	33	1.00	12	1.00
≤median	0	-	10	1.57 (0.84, 2.94)	4	0.98 (0.37, 2.62)	1	2.40 (0.33, 17.6)	0	-
>median	3	3.68 (1.16, 11.7)	4	0.65 (0.24, 1.74)	4	1.15 (0.43, 3.10)	1	3.63 (0.49, 26.7)	0	-
**Ever dust-10 y lag**										
No	87	1.00	493	1.00	315	1.00	33	1.00	12	1.00
Yes	3	1.50 (0.48, 4.76)	14	1.12 (0.66, 1.91)	8	1.06 (0.52, 2.14)	2	2.89 (0.69, 12.1)	0	-
**Lead Dust estimates - 10 y lag**										
No	87	1.00	493	1.00	315	1.00	33	1.00	12	1.00
≤median	0	-	10	1.57 (0.84, 2.94)	4	0.98 (0.37, 2.62)	1	2.40 (0.33, 17.6)	0	-
>median	3	3.68 (1.16, 11.7)	4	0.65 (0.24, 1.74)	4	1.15 (0.43, 3.10)	1	3.63 (0.49, 26.7)	0	-
**Ever dust-20 y lag**										
No	87	1.00	493	1.00	315	1.00	33	1.00	12	1.00
Yes	3	1.50 (0.48, 4.76)	14	1.12 (0.66, 1.91)	8	1.06 (0.52, 2.14)	2	2.89 (0.69, 12.1)	0	-
**Lead Dust estimates - 20 y lag**										
No	87	1.00	493	1.00	315	1.00	33	1.00	12	1.00
≤median	0	-	10	1.57 (0.84, 2.94)	4	0.98 (0.37, 2.62)	1	2.40 (0.33, 17.6)	0	-
>median	3	3.68 (1.16, 11.7)	4	0.65 (0.24, 1.74)	4	1.15 (0.43, 3.10)	1	3.63 (0.49, 26.7)	0	-

Exposure	Kidney cancer cases (N)	RR (95% CI)[a]	Lung cancer cases (N)	RR (95% CI)[a]	Stomach cancer cases (N)	RR (95% CI)[a]	Brain cancer cases (N)	RR (95% CI)[a]	Meningioma cases (N)	RR (95% CI)[a]
**Ever fume**										
No	81	1.00	460	1.00	293	1.00	33	1.00	12	1.00
Yes	9	1.62 (0.81, 3.24)	47	1.27 (0.94, 1.71)	30	1.39 (0.95, 2.03)	2	0.94 (0.23, 3.94)	0	-
**Lead Fume estimates**										
No	81	1.00	460	1.00	293	1.00	33	1.00	12	1.00
≤median	1	0.47 (0.07, 3.39)	12	0.98 (0.55, 1.74)	7	0.95 (0.45, 2.01)	1	1.23 (0.17, 9.01)	0	-
>median	8	2.34 (1.13, 4.86)	35	1.41 (1.00, 1.99)	23	1.62 (1.06, 2.48)	1	0.76 (0.10, 5.60)	0	-
**Ever fume-10 y lag**										
No	81	1.00	461	1.00	293	1.00	33	1.00	12	1.00
Yes	9	1.64 (0.82, 3.28)	46	1.25 (0.92, 1.69)	30	1.40 (0.96, 2.04)	2	0.95 (0.23, 3.98)	0	-
**Lead Fume estimates-10 y lag**										
No	81	1.00	461	1.00	293	1.00	33	1.00	12	1.00
≤median	1	0.48 (0.07, 3.47)	11	0.91 (0.50, 1.66)	7	0.97 (0.46, 2.05)	1	1.26 (0.17, 9.24)	0	-
>median	8	2.35 (1.13, 4.88)	35	1.41 (1.00, 1.99)	23	1.63 (1.06, 2.49)	1	0.77 (0.10, 5.62)	0	-
**Ever fume-20 y lag**										
No	82	1.00	462	1.00	296	1.00	33	1.00	12	1.00
Yes	8	1.51 (0.73, 3.14)	45	1.27 (0.93, 1.72)	27	1.30 (0.88, 1.93)	2	1.00 (0.24, 4.18)	0	-
**Lead Fume estimates-20 y lag**										
No	82	1.00	462	1.00	296	1.00	33	1.00	12	1.00
≤median	0	-	10	0.89 (0.47, 1.66)	6	0.88 (0.39, 1.98)	1	1.35 (0.18, 9.90)	0	-
>median	8	2.41 (1.16, 5.01)	35	1.44 (1.02, 2.04)	21	1.51 (0.97, 2.35)	1	0.79 (0.11, 5.81)	0	-

[a]Adjusted for education, income level, and lifetime pack-years of cigarette use.
